# Emotional exhaustion and unhealthy eating among COVID-19 front-line healthcare workers during recuperation: A cross-sectional study

**DOI:** 10.3389/fpubh.2022.926395

**Published:** 2022-08-25

**Authors:** Wei Yan, Xinyao Zhou, Caiping Song, Xu Luo, Huan Wang, Pengpeng Yin, Hao Wu, Junying Ye

**Affiliations:** ^1^School of Economics and Business Administration, Chongqing University, Chongqing, China; ^2^Economics and Management School, Wuhan University, Wuhan, China; ^3^Xinqiao Hospital, Army Medical University, Chongqing, China; ^4^Department of Medical Administration, Southwest Hospital, Army Medical University, Chongqing, China; ^5^Development and Planning Department, Chongqing Medical University, Chongqing, China; ^6^Department of Scientific Research and Education, Chongqing Health Center for Women and Children, Women and Children's Hospital of Chongqing Medical University, Chongqing, China

**Keywords:** affective rumination, emotional exhaustion, unhealthy food consumption, COVID-19, front-line healthcare workers

## Abstract

**Objective:**

Thousands of healthcare workers on the frontlines who have been battling the COVID-19 pandemic could face emotional and mental health risks even after their critical pandemic work. This study examined the impact of affective rumination on emotional exhaustion and the spillover effect of affective rumination on unhealthy food consumption among healthcare workers during recuperation.

**Methods:**

A total of 418 frontline healthcare workers from 10 Chinese medical institutions were recruited through random cluster sampling. A linear mixed model in SPSS25.0 was performed for hierarchical regression to analyze the effect of affective rumination on unhealthy food consumption via emotional exhaustion. A conditional process analysis was employed to investigate the moderating role of family support in the mediating effect of emotional exhaustion.

**Results:**

Front-line healthcare workers scored at a medium level on an emotional exhaustion scale (2.45 ± 0.88). Affective rumination mediated by emotional exhaustion had a significant positive predictive effect on unhealthy food consumption. The indirect effect accounted for ~43.9% of the total effect. Family support amplified the effect of emotional exhaustion on unhealthy food consumption (β = 0.092, *p* < 0.05).

**Conclusion:**

Affective rumination could be a cause of emotional exhaustion and unhealthy food consumption. First-line healthcare workers could be screened for possible emotional exhaustion through the evaluation of affective rumination in order to provide them with targeted interventions. Family support did not prove to be beneficial in all cases as it enhanced the positive effect of emotional exhaustion on unhealthy eating in the current study. Therefore, family support should be carefully integrated in future interventions.

## Graphical Abstract

In this study, we examined the impact of affective rumination on emotional exhaustion and the spillover effect of affective rumination on unhealthy food consumption among healthcare workers during recuperation. Emotional exhaustion was an important mediator of the relationship between affective rumination and unhealthy food consumption. Interestingly, family support did not prove to be beneficial in all cases, as it enhanced the positive effect of emotional exhaustion on unhealthy eating in the current study. 
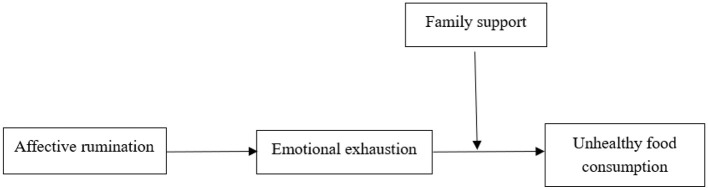

## Highlights

- Affective rumination is an important cause of increased emotional exhaustion among frontline healthcare workers.
- Emotional exhaustion is an important mediator in the relationship between affective rumination and unhealthy food consumption.
- Family support amplified the effect of emotional exhaustion on unhealthy food consumption.
- Front-line healthcare workers showed a medium level of emotional exhaustion, suggesting that some of the negative impacts of their work battling COVID-19 were long lasting.
- Interventions designed to support healthcare workers during a public health crisis should focus on the role of family members, and family support should be carefully integrated into future interventions.

## Introduction

COVID-19 is the fastest spreading, most extensive, and most challenging public health emergency in history. The impacts of this pandemic represent a huge burden and have provoked unimaginable losses across the globe (1). Front-line healthcare workers experience exceptionally high rates of physical burnout, mental stress, occupational risk of infection, and increased risk of morbidity and mortality (2). Emotional exhaustion is a chronic state of physical and emotional depletion resulting from excessive work, overwhelming personal demands, and/or continuous stress. It has been observed that medical workers experience a higher level of emotional exhaustion during the COVID-19 outbreak (3–6). Existing studies have shown that emotional exhaustion affects both the physical and mental health and work efficiency of medical personnel as well as the quality of medical and health services (7). Healthcare workers are at risk of developing PTSD after deployment to confront a public health emergency (8) and are prone to serious long-term mental distress. It is important to study the ways in which medical workers have been affected by the COVID-19 outbreak to determine how emotional exhaustion may have led to excessive negative impacts on work and life and to understand whether social support could have buffered these effects.

China's initial response to COVID-19 provided a useful example of the effective management of the crisis (9). At the beginning of the COVID-19 outbreak, more than 40,000 healthcare workers from different cities were deployed in Hubei to fight the epidemic (10). This mobilization of healthcare personnel played an important role in controlling epidemic emergencies. Therefore, it is reasonable to believe that frontline healthcare workers experienced tremendous physical and psychological stress during their recuperation after their mission to Hubei Province. Previous studies have revealed the causes, mechanisms, and interventions related to the emotional exhaustion of healthcare workers during the COVID-19 outbreak (11, 12), however, few studies have examined whether frontline workers who fought against COVID-19 experienced emotional exhaustion and other health problems after that work ended.

Unhealthy food consumption is a behavioral indicator of employees' lack of self-regulation and maladjustment (13). Poor eating habits also serve as an indicator of how negative work experiences may spill over into non-work areas. According to Adler's individual psychology theory, people may unconsciously seek alternative means to alleviate anxiety in a negative mood (14). Previous studies have shown that the eating habits of healthcare professionals are affected during the new crown epidemic, reducing physical activity and exercise, increasing carbohydrate and alcohol intake, and increasing the possibility of weight gain and immune damage (15). Some first-line nurses who fight COVID-19 have obvious negative emotions, such as depression and anxiety, and the quality of their diet and sleep are poor (16). Moreover, there was evidence that first-line medical staff who fought COVID-19 had a low level of nutrition knowledge, lack of attention to nutrition and health, and a low score for healthy diet collocation (17). The above studies suggest that the dietary behaviors of frontline medical staff might be affected by the epidemic. However, no follow-up study has been conducted to explore whether the experience of fighting COVID-19 will affect the work and life of frontline medical staff even after the emergency mission. Front-line healthcare workers could have been more likely to turn to junk food, binge eating, or other unhealthy behaviors to alleviate negative emotions after working in a stressful environment for a long period (18). Moreover, there is a tradition of celebrating banquets with friends and relatives in Chinese culture. We surmised that these conditions would lead to unhealthy diets among frontline doctors. This study focuses on the spillover effect of emotional exhaustion among frontline healthcare workers during recuperation. We introduced an index of unhealthy food consumption to explore the impact of emotional exhaustion on a healthy diet and to reveal the impact of family support on workers' moods and behaviors. This study on unhealthy food consumption permitted an analysis of the spillover effect of emotional exhaustion, as well as a means by which to gauge the scope of influence of rumination during a public health emergency. Additionally, this study addressed the trajectory of emotional exhaustion, including spillover, from family factors. This research was designed to increase the understanding of healthcare workers' exhaustion and contribute to the improvement of human resource management practices during major public health emergencies.

## Research hypotheses

### Affective rumination and emotional exhaustion

Affective rumination is characterized by intrusive and recurrent negative thoughts that are usually related to work attitudes and behaviors. This type of rumination involves activation of the sympathetic nervous system (19). Effort-Recovery Theory addresses how prolonged or repeated daily stress at work (effort) may adversely affect health if not balanced by sufficient recovery (20). Engagement in non-work' activities (low-effort, social, and physical activities) results in higher daily recovery, which involves the current emotional state, causes, consequences, and meaning of the event that led to this state. It is believed to be an important factor in the development of depression, impaired physical and mental health, and an increase in work-related burnout (21–23). Numerous studies have also emphasized the positive mediating role of rumination in emotional exhaustion (24). Affective rumination continuously consumes cognitive and emotional resources. When individuals ruminate affectively, they often feel upset and anxious because of their work. This cognitive presentation of workplace stressors not only fails to help secure the resources needed but also consumes resources. Consequently, an individual's ability to recuperate following a stressful event is hindered and the corresponding level of emotional exhaustion remains elevated. Based on these studies, the following hypothesis is proposed:

H1: Affective rumination positively affects emotional exhaustion.

### Affective rumination and unhealthy food consumption

Many healthcare workers suffer from nutritional and health problems due to irregular lifestyles, unhealthy food consumption, and high levels of stress in work and life (25). A significant proportion of people tend to rely on excessive food consumption to vent their stress and negative emotions, resulting in cardiovascular disease, excessive weight or obesity, hyperlipidemia, high blood pressure, and other diseases (26). Front-line healthcare workers are under enormous pressure during the COVID-19 pandemic. Therefore, it is important to consider unhealthy food consumption. We hypothesized that the repetition of negative events and emotions during rumination may lead healthcare workers to alleviate the pain of negative emotions through the consumption of unhealthy food.

H2: Affective rumination is positively related to unhealthy food consumption.

### The mediating role of emotional exhaustion

Emotional exhaustion is a chronic state of physical and emotional depletion caused by excessive work, personal demands, or continuous stress (27). When healthcare workers' emotional exhaustion level is relatively high, they are likely to have an unhealthy lifestyle due to a lack of psychological cognitive resources. According to a survey conducted in 2018, many healthcare workers engage in unhealthy diets because of work pressure, overtime, and depression (28). When workers engage in rumination, their level of emotional exhaustion increases, which may lead to unhealthy food consumption. Therefore, we propose the following hypothesis:

H3: Affective rumination mediated by emotional exhaustion has a positive effect on unhealthy food consumption.

### The moderating role of family support

Family support, a resource provided by family members, has been shown to have an important influence on individual development and adaptation (29). Studies have demonstrated that positive family relationships help individuals overcome negative mental states such as anxiety and depression. Healthcare workers with sufficient family support remained confident and strong in dealing with setbacks. Wu et al. (30) collected data on 397 students two and a half years after the Yaan earthquake in Sichuan, China. According to his findings, social support moderated the relationship between hyperarousal, avoidance, PTSD, and life satisfaction. Song et al. (31) surveyed 497 nurses at Peking Union Medical College Hospital and found that social support moderated the relationship between burnout and depression, especially between the dimensions of exhaustion and depression. Front-line healthcare workers' emotional exhaustion increases when they experience repetitive flashbacks of negative events or emotions. When the level of family support was high, it mitigated the need for personal resources and reduced the impact of affective rumination and emotional exhaustion on the quality of life. In summary, our hypotheses are as follows.

H4: Family support buffers the effect of emotional exhaustion on unhealthy food consumption. In addition, higher levels of family support corresponded to a lower indirect effect of affective rumination on unhealthy food consumption *via* emotional exhaustion.

A theoretical model was established based on the aforementioned hypotheses, as shown in Graphical Abstract.

## Methods

### Participants and procedure

The study was conducted in Chongqing, China. On March 29, 2020, all medical teams in Chongqing province supporting Wuhan finished the task and returned. After 14 days of isolation, they returned to their original units in mid-April. After a recovery period of ~3 months, we began the study on July 12. Cluster random sampling was conducted to recruit healthcare workers who were once at the frontline of Wuhan during the COVID-19 pandemic. Using the lottery method, Four of the 18 sent to Wuhan from Chongqing for assistance (the third medical team of Chongqing province, the ninth medical team of Chongqing province, the first medical team of the Army Military Medical University, and the second medical team of the Army Military Medical University) were selected to participate in this study. The inclusion criteria were as follows:

1) worked as frontline health workers and participated in the fight against COVID-19 in Hubei province;
2) able to read and complete the questionnaires independently;
3) volunteered to participate in this study.

We administered the survey online to avoid unnecessary human contact *via* WeChat social media. If the participants did not answer the current question, the WeChat applet could not jump to the next question. We depended on IP addresses to identify and eliminate duplicate participants. The questionnaire used was piloted with a small number of participants to ensure the accuracy of the expression of content. The four questionnaires were not changed after the pilot study, and the data of the pilot study were combined with the final sample.

This study was conducted between July 12 and July 20, 2020, with a total of 418 healthcare workers. We did not include people who were recently affected by major events other than COVID-19, or those with a history of neurasthenia or trauma. The demographic characteristics of the participants are presented in Table 1.

**Table 1:** Demographic characteristics (*N* = 418).

**Variable**	***N***	**%**
Gender
Male	125	29.90%
Female	293	70.10%
Age (year)
<20	0	0.00%
20–29	93	22.25%
30–39	252	60.29%
40–49	68	16.27%
>50	5	1.20%
Education
College or under	37	8.85%
Undergraduate	311	74.40%
Master	41	9.81%
Doctor	29	6.94%

This study was approved by the Ethics Committee of the Chongqing Maternal and Child Health Hospital. The participants provided informed consent before participating in the study.

### Measures

A back-translation procedure was conducted to ensure the accuracy and consistency of the translated scales (32). A bilingual management professor translated the items into Chinese from the original English, and another management professor translated the items from Chinese back into English. These scales have been widely accepted in academic research. All variables were scored on a five-point Likert scale ranging from 1 (strongly disagree) to 5 (strongly agree). The variables and instruments used to measure them are as follows:

Affective rumination was measured by a five-item scale developed by McCullough et al. (33). We included the battle against COVID-19 in the survey to capture events relevant to the participants. The scale included such items as “during my free time, I become tense by thinking about issues related to the work against COVID-19” and “I am annoyed when I think about issues related to the work to fight COVID-19.” The scale coefficient (α) for internal consistency was 0.872.

Emotional exhaustion was measured using the five-item scale (MBI-GS) developed by Maslach et al. (34). Sample items included “I feel exhausted from work.” and “I'm about to experience work burnout.” The scale coefficient (α) for internal consistency was 0.919.

Family support was measured using the four-item scale developed by Procidano and Heller (35). The scale included items such as “My family can provide me with help” and “I can talk to my family about my problems.” In this study, the scale coefficient (α) for the internal consistency was 0.911.

Unhealthy food consumption was measured using a four-item scale developed by Liu et al. (36). Sample items included “today I ate too much junk food after work” and “today I had too many late-night snacks before going to bed.” The scale coefficient (α) for internal consistency was 0.878.

### Analytical approach

We conducted a confirmatory factor analysis (CFA) of the questionnaire with MPLUS 8, hierarchical regression analysis with SPSS 25.0, and further testing, such as conditional process analysis with PROCESS 3.3. We standardized the independent, mediating, and moderating variables to avoid multicollinearity caused by the addition of interactions. Affective rumination was used as the predictive variable, emotional exhaustion as the mediating variable, family support as the moderating variable, and emotional exhaustion as the outcome variable.

### Quality control

All study variables were self-reported by participants and were collected within a specific period, which may have caused bias by common method variance (CMV). Therefore, we conducted a Harman's single-factor test to measure the degree of bias in the current study. The SPSS results showed that five factors were generated when the data were not rotated, explaining 71.4% of the variation. The first principal component was 29.6%, revealing 29.6% explanatory power of the variance for all questions, which did not exceed the 50% judgment criteria. Therefore, common method variance was not a major concern in this study and the research results had reasonable reliability.

## Results

### Reliability and validity

The scores reflecting internal consistencies for the variables were all over 0.85, which reflected the acceptable reliability of the measuring instruments. The discriminant validity between variables was measured using confirmatory factor analysis (CFA). As shown in Table 2, the four-factor model was superior to the other models in that it presented the best fit for the data (χ^2^/df = 9.80, RMSEA = 0.10, TLI = 0.87, CFI = 0.90, SRMR = 0. 07). The variables proved to have reasonable validity and the data were not influenced by homologous bias.

**Table 2:** Confirmatory factor analysis (*N* = 232).

**Model**	**Factor**	**χ^2^**	**df**	**χ^2^/df**	***Δχ* ^2^**	**TLI**	**CFI**	**RMSEA**	**SRMR**
1	Four-factor	1264.48	129	9.80		0.869	0.890	0.101	0.071
2	Three-factor	1295.40	132	9.81	30.92	0.725	0.763	0.145	0.129
3	Two-factor	2008.63	134	14.99	713.23	0.564	0.618	0.183	0.145
4	One-factor	2998.69	135	22.21	990.06	0.339	0.416	0.225	0.177

Four-factor model: affective rumination, emotional exhaustion, unhealthy food consumption, family support; three-factor model: affective rumination + unhealthy food consumption, emotional exhaustion, family support; two-factor model: affective rumination + emotional exhaustion + unhealthy food consumption, family support; one-factor model: affective rumination + emotional exhaustion + unhealthy food consumption + family support.

### Descriptive statistics and correlations

Table 3 presents the descriptive statistics and correlations between the variables. Affective rumination positively related to emotional exhaustion (*r* = 0.392, *p* < 0.01) and unhealthy food consumption (*r* = 0.199, *p* < 0.01). Emotional exhaustion was positively associated with unhealthy food consumption (*r* = 0.306, *p* < 0.01). Emotional exhaustion was negatively related to family support (*r* = −0.303, *p* < 0.01).

**Table 3:** Descriptive statistics and correlations (*N* = 418).

**Variables**	**Mean**	**SD**	**Affective**	**Emotional**	**Unhealthy**	**Family**
	**rumination**	**exhaustion**	**food consumption**	**support**
Affective rumination	1.68	0.65	1	0.392**	0.199**	−0.158**
Emotional exhaustion	2.45	0.88	0.392**	1	0.306**	−0.303**
Unhealthy food consumption	2.19	0.87	0.199**	0.306**	1	−0.277**
Family support	4.03	0.81	−0.158**	−0.303**	−0.277**	1

*p < 0.05, **p < 0.01 (two-tailed).

### Indirect effect of emotional exhaustion

Hierarchical linear regression analysis was used to test the hypotheses. Table 4 illustrates that affective rumination is positively related to emotional exhaustion (β = 0.626, *p* < 0.001) and unhealthy food consumption (β = 0.228, *p* < 0.001).

**Table 4:** Indirect effect of emotional exhaustion (*N* = 418).

**Variables**	**Unhealthy food**			**Emotional**	
	**consumption**			**exhaustion**	
	**Step 1**	**Step 2**	**Step 3**	**Step 4**	**Step 5**
	**(β)**	**(β)**	**(β)**	**(β)**	**(β)**
Gender	0.018	0.057	0.009	0.128	0.198
Age	−0.352***	−0.381***	−0.338***	−0.121	−0.172*
Education	−0.007	−0.011	−0.049	0.162*	0.154*
Affective rumination		0.228***	0.128*		0.626***
Emotional exhaustion			0.246***		
R^2^	0.055	0.106	0.156	0.014	0.177
ΔR^2^	-	0.051	0.101	-	0.163
F	8.067***	23.61***	24.379***	1.959	81.564***

*p < 0.05, **p < 0.01, ***p < 0.001 (two-tailed); gender, age, and education served as control variables; and affective rumination and emotional exhaustion served as predictive variables.

Emotional exhaustion partially mediated the relationship between affective rumination and unhealthy food consumption. The regression coefficient for affective rumination decreased when emotional exhaustion was included (β = 0.228 to β = 0.128, *p* < 0.001 to *p* < 0.05).

We also performed 5,000 bootstraps for deviation correction to verify the mediating effect. As shown in Table 5, the indirect effect of emotional exhaustion was 0.1, and the confidence intervals (CI) were 0.058 and 0.146, which did not include a value of 0. Therefore, emotional exhaustion has a significant mediating effect on the relationship between affective rumination and unhealthy food consumption. The direct effect was also significant (CI = 0.028, 0.225), indicating that emotional exhaustion had a partial mediating effect.

**Table 5:** Bootstrap test for mediating effect (*N* = 418).

**Mechanism**		**Effect**	**Boot**	**Boot**	**Boot**
			**SE**	**LLCI**	**ULCI**
AR → EE → UFC	Indirect effect	0.100	0.023	0.058	0.146
	Direct effect	0.128	0.049	0.028	0.225
	Total effect	0.228	0.046	0.136	0.317

AR, affective rumination; EE, emotional exhaustion; UFC, unhealthy food consumption; bootstrap is set by a 95% confidence interval for 5,000 repeated samples.

### Moderating influence of family support

Hierarchical linear regression was employed to test the moderating effect of family support.

Table 6 illustrates the moderating effect of family support. Contrary to our hypothesis, family support enhanced the effect of emotional exhaustion on unhealthy food consumption (β = 0.092, *p* < 0.05).

**Table 6:** Moderating influence of family support (*N* = 418).

**Variables**	**Unhealthy food consumption**		
	**Step 1 (**β**)**	**Step 2 (**β**)**	**Step 3 (**β**)**
Gender	0.018	0.009	0
Age	−0.352***	−0.303***	−0.303***
Education	−0.007	−0.032	−0.036
Emotional exhaustion		0.24***	0.24***
Family support		−0.188***	−0.197***
Emotional exhaustion × Family support			0.092*
R^2^	0.055	0.174	0.183
ΔR^2^	–	0.119	0.008
F	8.067***	29.745***	4.218*

*p < 0.05, **p < 0.01, ***p < 0.001 (two-tailed).

**Figure 1:**
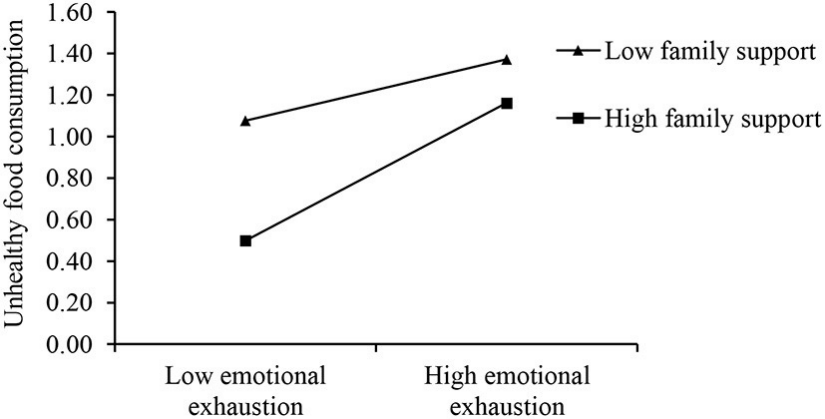
Moderating influence of family support (*N* = 418).

As depicted in Figure 1, these results revealed that family support interacted with emotional exhaustion. The positive relationship between emotional exhaustion and unhealthy food consumption was stronger when family support was high.

We further tested the moderating effect on the indirect path using a conditional process analysis. We identified three different levels of family support that explain the different levels of significance of the indirect effect of affective rumination on unhealthy food consumption through emotional exhaustion (Table 7). Specifically, the indirect effect of affective rumination on unhealthy food consumption was stronger when family support was higher (effect = 0.118, CI = 0.057, 0.180) rather than lower (effect = 0.040, CI = −0.021, 0.104). Therefore, family support moderates the indirect effect of affective rumination on unhealthy food consumption through emotional exhaustion.

**Table 7:** Conditional process analysis.

**Family support**	**Effect**	**BootSE**	**BootLLCI**	**BootULCI**
Low level	0.040	0.032	−0.021	0.104
Moderate level	0.079	0.022	0.036	0.125
High level	0.118	0.031	0.057	0.180

Bootstrap is set at a 95% confidence interval for 5,000 repeated samples. BootSE, bootstrap standard error; BootLLCI, lower limit of the bootstrap confidence interval; BootULCI, upper limit of the bootstrap confidence interval.

## Discussion

Since the emergence of a coronavirus disease in December 2019, healthcare workers have been bearing more serious work and psychological burdens, which has brought great challenges to the health of medical staff (37, 38). Several previous investigations have suggested that mental health issues have shown a rapid increase among frontline healthcare workers during the COVID-19 pandemic, including anxiety, depression, emotional exhaustion, PTSD (39, 40). This study focused on the emotional and mental threats faced by frontline healthcare workers during the period following their deployment to fight COVID-19 in Wuhan, China. We explored the effects of affective rumination on emotional exhaustion. We found that affective rumination could be a cause of emotional exhaustion and unhealthy food consumption during the recuperation period. Moreover, family support appeared to aggravate the spillover effect of emotional exhaustion. Several findings merit attention.

First, the emotional exhaustion levels of frontline healthcare workers during recuperation were lower than those at the peak of the COVID-19 pandemic (41). This discrepancy could be explained by the possibility that the workers had a chance to recover following a period of high-intensity work with COVID-19 patients, and their emotional exhaustion levels returned to normal. According to our research, the emotional exhaustion of frontline healthcare workers was at a medium level, suggesting that some of the negative impacts of their work battling COVID-19 were long-lasting. Yuan's research on healthcare workers from seven cities in China revealed that those between the ages of 20 and 29 years engaged in unhealthy food consumption (42). We also found that subjects between the ages of 30 and 39 years (60% of the total sample) maintained healthy eating habits even when experiencing negative emotions.

Second, affective rumination mediated by emotional exhaustion had a positive predictive effect on unhealthy food consumption. This finding is consistent with that of Boren (43) and Vandevala et al. (44). Affective rumination is an important cause of increased emotional exhaustion among frontline healthcare workers. Negative events and emotions emerged repeatedly at the cognitive level during rumination, which demanded the expenditure of psychological and emotional resources. Emotional exhaustion was an important mediator of unhealthy food consumption caused by affective rumination, and its indirect effect accounted for 43.9% of the total effect. Although studies have shown that many people eat unhealthy foods that are high in calories, fat, and sugar when they are stressed (45), there has been little discussion on how affective rumination affects unhealthy food consumption.

Our study identified two pathways by which affective rumination affected unhealthy food consumption. First, rumination exacerbates stress. The constant presence of negative events and emotions at the cognitive level during rumination consumes healthcare workers' psychological and emotional resources. They would turn to unhealthy foods to relieve stress. Second, affective rumination results in high levels of emotional exhaustion. Some participants lacked the psychological resources necessary to regulate their lifestyle and, consequently, were more likely to indulge in unhealthy food consumption.

Finally, contrary to our hypothesis, family support amplified the impact of affective rumination on unhealthy food consumption through emotional exhaustion. Family support is often considered to have health benefits similar to those of positive thinking (46). Family support could help individuals who are under high pressure at work cope with stress and emotional exhaustion and boost their self-confidence. The results of our study indicated that family support was negatively associated with unhealthy food consumption; however, this effect was not pronounced for those who experienced high levels of emotional exhaustion. Frequent family interactions can exacerbate burnout and stress, which can lead to exhaustion and even impact other areas of life (47). Front-line healthcare workers tend to have frequent interactions with family members if family support is strong. The constant reminder of their negative experiences in conversations with the family could result in excessive expenditure on psychological resources. The elevated level of resources needed for these interactions could result in an attempt to regulate emotions through excessive or unhealthy food consumption. Therefore, there is a need to further explore the mechanism of family support, and not assume that the existence of social support proves helpful in all circumstances.

Some immediate practical implications are derived from this study's results. First, there is a need to attend to front-line healthcare workers' psychological and emotional health, especially during pandemics. In particular, workers with a history or currently are at risk of emotional exhaustion could also be screened for affective rumination as a means by which to initiate timely interventions.

Second, interventions designed to support healthcare workers during crises should focus on the role of family members. The findings from this study suggest that it is imperative to educate the families of healthcare workers about the ways that a focus on the crisis without offering solutions could result in greater harm than good. Appropriate interventions for the entire family can help front-line workers cope with affective rumination. Finally, the development of appropriate courses for family members can help frontline healthcare workers cope with affective rumination, thereby enhancing the effectiveness of family support.

## Limitations

Our study has two limitations. First, there was a possibility of same-source bias because the study was cross-sectional, and all items were self-reported. Although confirmatory factor analysis in this study proved that the problem of common method variance was not significant and the results were reliable, it is suggested that future research should include data with multiple time points and sources to ensure the validity and reliability of surveys and to further explain the relationship among variables. Second, the study participants were limited to Chinese healthcare workers. Therefore, these results may not be applicable to other populations. In China, rumination is regarded as a positive behavior that promotes self-growth through introspection and reflection on past experiences. However, due to the differences in history and culture, the affective rumination level of healthcare workers in other countries may be higher than that in China. Future research could expand its scope of application by collecting samples from different countries and populations.

## Conclusion

Affective rumination could be a cause of emotional exhaustion and unhealthy food consumption. First-line healthcare workers could be screened for possible emotional exhaustion through the evaluation of affective rumination in order to provide them with targeted interventions. Family support did not prove to be beneficial in all cases as it enhanced the positive effect of emotional exhaustion on unhealthy eating in the current study. Therefore, family support should be carefully integrated in future interventions.

## Data availability statement

The raw data supporting the conclusions of this article will be made available by the authors, without undue reservation.

## Ethics statement

The studies involving human participants were reviewed and approved by the Ethics Committee of Chongqing Maternal and Child Health Hospital. Written informed consent for participation was not required for this study in accordance with the national legislation and the institutional requirements.

## Author contributions

WY and XZ contributed to the design and analysis, writing, and revision. CS and XL contributed to data collection and data analysis. HWa and PY contributed to the writing of the paper. HWu and JY contributed to data collection and the writing of the article. All authors read and approved the final manuscript.

## Funding

This research was supported by the grants funded by National Social Science Foundation of China (19BJY052), National Natural Science Foundation of China (72110107002 and 71974021), and Natural Science Foundation of Chongqing (cstc2021jcyj-msxmX0689).

## Conflict of interest

The authors declare that the research was conducted in the absence of any commercial or financial relationships that could be construed as a potential conflict of interest.

## Publisher's note

All claims expressed in this article are solely those of the authors and do not necessarily represent those of their affiliated organizations, or those of the publisher, the editors and the reviewers. Any product that may be evaluated in this article, or claim that may be made by its manufacturer, is not guaranteed or endorsed by the publisher.
